# Protective effect of mandatory face masks in the public—relevant variables with likely impact on outcome were not considered

**DOI:** 10.1073/pnas.2012415117

**Published:** 2020-10-13

**Authors:** Günter Kampf

**Affiliations:** ^a^Institute for Hygiene and Environmental Medicine, University Medicine Greifswald, 17475 Greifswald, Germany

Zhang et al. (1) conclude that wearing a face mask in public is the most effective means to prevent transmission. This conclusion is scientifically highly questionable. First, the number of epidemic-curve examples is small; an explanation of how they were chosen is lacking. Second, the evaluation is flawed by not taking into account where the majority of transmissions took place locally (e.g., in the public or by healthcare workers) and if adequate personal protective equipment was available for healthcare workers (2). Third, the authors assumed that face covering was the only effect and did not control for or analyze confounding variables. It is very unlikely that “social distancing” was the same in all selected epicenters. The World Health Organization recommends at least 1-m distance (3), whereas the Centers for Disease Control and Prevention recommends 6 feet (∼2 m). It is obvious that the distance itself is likely to have an impact on transmission. Would physical distancing be as effective as face masks when a distance >2 m would be the global standard? This important variable is not included for Italy, China, or the United States. Fourth, weather conditions or the population density may have an impact on its own (4). Coronavirus infections are usually seasonal infections resulting in a flattened curve toward the summer anyway (5). The different epidemic curves for the United States and New York shown by the authors may be also explained by differences of seasonality for New York alone and the entire United States including southern states where the epidemic arrived later. Fifth, mandatory face masks in the public may have the effect that fewer people leave their homes, resulting in a lower population density in the public followed by lower transmission rates. Face masks have been described to increase physical distancing in front of shops (6). However, Zhang et al. do not provide any observational data to demonstrate that population densities or distances were similar in each epicenter before and after mandatory face masks. Sixth, the authors claim that mandated face covering "significantly reduces the number of infections." This claim may be wrong because all databases count “cases” based on the nasopharyngeal detection of severe acute respiratory syndrome coronavirus 2 (SARS-CoV-2) RNA (7). A case is not necessarily a clinical infection because a substantial proportion of SARS-CoV-2 RNA carriers remain asymptomatic (8). Seventh, multiple interventions may have been implemented simultaneously, so that the differences are not necessarily attributable to just masks alone. Finally, data from Germany indicate that mandatory face masks in shops and public transport as a single measure did not accelerate the decline of new cases (9). The effect of any measure should have a suitable control including a stratification regarding the most relevant parameter such as age and health of population, epidemic stage, population density, season, weather, and compliance with the intervention measured by observation. The controls are lacking so that the authors’ assumptions are insufficiently justified, and therefore their analysis does not support their main claim.
